# Detection and Identification of VOCs Using Differential Ion Mobility Spectrometry (DMS)

**DOI:** 10.3390/molecules27010234

**Published:** 2021-12-30

**Authors:** Wojciech Fabianowski, Mirosław Maziejuk, Monika Szyposzyńska, Monika Wiśnik-Sawka

**Affiliations:** 1 Military Institute of Chemistry and Radiometry, Al. gen. Antoniego Chruściela “Montera” 105, 00-910 Warsaw, Poland; w.fabianowski@wichir.waw.pl (W.F.); m.szyposzynska@wichir.waw.pl (M.S.); m.wisnik-sawka@wichir.waw.pl (M.W.-S.); 2 Faculty of Advanced Technologies and Chemistry, Military University of Technology, ul. gen. Sylwestra Kaliskiego 2, 00-908 Warsaw, Poland

**Keywords:** volatile organic compounds, differential ion mobility spectrometry, ion mobility spectrometry

## Abstract

The article presents a technique of differential ion mobility spectrometry (DMS) applicable to the detection and identification of volatile organic compounds (VOCs) from such categories as n-alkanes, alcohols, acetate esters, ketones, botulinum toxin, BTX, and fluoro- and chloro-organic compounds. A possibility of mixture identification using only the DMS spectrometer is analyzed, and several examples are published for the first time. An analysis of different compounds and their mechanisms of fragmentation, influence on effective ion temperature, and high electric field intensity is discussed.

## 1. Introduction

Volatile organic compounds (VOCs) are always present in the atmosphere. They originate mostly from liquids, and also from solids. Unfortunately, they can have a negative impact on human health. VOCs originating from human industrial activity cause air pollution, which can be dangerous. This is a problem with which inhabitants of areas surrounding chemical plants, coal mines, or cities with heavy traffic are familiar [1]. VOCs pose a risk to health even indoors, where they can be found in paints, washing-up liquids, aerosols, glues, and even burning candles [2,3]. While some VOCs are mere irritants because of their unpleasant smell, others may cause serious health problems and, thus, should be closely monitored. This is especially important in high-traffic areas, such as airports, railway stations, or clubs, where it is not only recommended, but also required to monitor air quality. There are various techniques to measure VOCs and, therefore, to control their content in air—especially in order to detect dangerous materials such as narcotics or explosives.

Several methods are used for VOC control, including the most popular and widely applied electronic e-noses (sensor arrays), which work in dry or humid air conditions [4,5]; MOX sensors, with VOC measurement accuracy at the ppb level [6,7]; very promising surface acoustic wave (SAW) sensors with different polymer coatings [8], or with a thin polymer layer with embedded solid piezoelectric ZnO particles or magnetic Fe_3_O_4_ particles [9,10], or even carbon nanotube (CNT) particles [11,12]; and quartz crystal microbalance (QCM) sensors with a thin pentacene layer for the detection of aromatic hydrocarbons [13,14]. These semiconductor instruments are widely used for monitoring air conditioning systems, but they suffer from pure resolution. The following methods are more sensitive, but also more costly: high-performance liquid chromatography (HPLC) [15]; gas chromatography (GC) and gas chromatography combined with mass spectrometry (GC/MS) [16,17,18,19,20,21,22,23,24,25]; infrared spectroscopy (IR); and Fourier-transform infrared spectroscopy (FTIR) [26,27,28,29].

Detection of hazardous substances in the air at levels well below the threshold limit values (TLVs) is becoming more and more important. One such technique widely used for air monitoring is ion mobility spectrometry (IMS). The main topic of this research is testing the similar technology differential ion mobility spectrometry (DMS) for the detection of various types of VOCs in the air. This article shows that this technology can potentially be used for monitoring air pollution. This technology guarantees high-speed detection (reaction time as low as <10 s), high sensitivity, and recognition of the types of dangerous gases in the monitored air.

## 2. Materials and Methods

**Figure 1:**
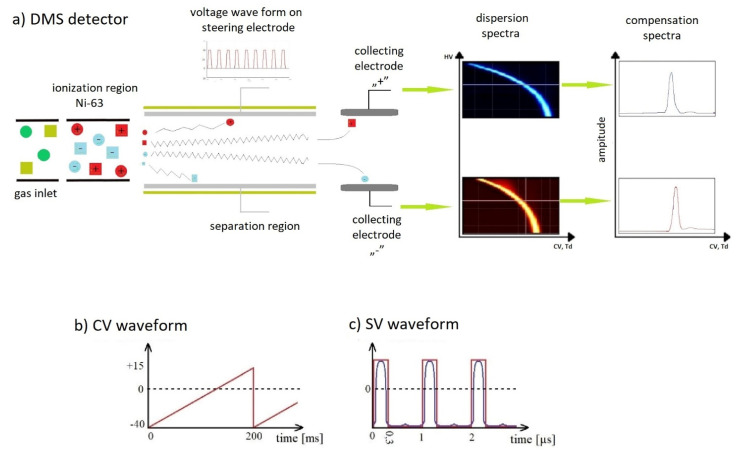
Scheme of a differential ion mobility spectrometer: (**a**) general scheme of the DMS spectrometer, and routes of ionized positive and negative species; (**b**) CV waveform; (**c**) SV waveform.

Differential ion mobility spectrometer (DMS) (Figure 1) chamber construction consists of two parallel electrodes placed very close to one another, usually within a distance of 0.25 mm to 1.5 mm.

In the DMS detector, an introduced carrier gas and a sample are ionized with the Ni-63 ionizing source. Next, the carrier gas and the sample enter the separation region, where ions are separated by the influence of the changing electric field. This field is created by applying changing voltage to one of the electrodes. This voltage is the sum of a slow-varying saw-tooth waveform with a frequency of a few hertz (compensation voltage; CV) and a quasi-rectangular waveform voltage with a duty cycle of ~30% and a frequency of ~1 MHz (separation voltage; SV). The second electrode is held at zero potential. The field gradient between the electrodes is very high, and usually exceeds 15 kV cm^−1^. The voltage generator used in the system should enable voltage regulation within the range of 0.5–2 kV. Compensation voltage and separation voltage are usually expressed in Td units—reduced electric field (E/N, where E is the electric field intensity and N is the number of molecules), which can be calculated with the following formula: 1 Td = 10^−17^ V cm^2^. Under standard conditions (1 atm, 0 °C), N_0_ = 2.687 × 10^19^ cm^−3^, so 1 Td corresponds to 268.7 V/cm [30].

**Figure 2:**
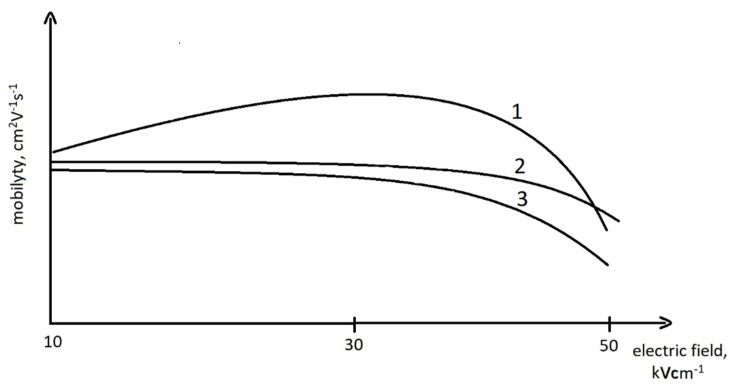
Dependence of ions’ mobility on electric field tension within the DMS detector. 1: common behavior of ions; 2 and 3: behavior of heavy ions and dimers, respectively.

Ions’ mobility may change under the influence of the changing electric field. Changes in ions’ mobility parameters are specific to the examined ions. The change in mobility versus the electric field in the DMS detectors, as described by B. B. Schneider [31], is presented in Figure 2.

Figure 2 presents different modes of behavior of the ion mobility coefficient in relation to the electrical field. For curve #1 (the most common behavior of ions), ions’ mobility increases at the beginning and then decreases. For curves #2 and #3 (usually ions of heavy particles, dimers, or trimers), the change in ions’ mobility in the starting period is not significant, and then it decreases decisively. The speed of these changes varies for different ions.

The range of ions’ mobility depends on their size, shape, and charge. The analyte ions form cluster ions with neutral molecules in the carrier gas.

Ions’ mobility increases with increasing electric field when the number of neutral particles connected with the ions decreases. However, when the number of neutral particles is the same and the ions’ energy increases, their mobility decreases. In the case of DMS, the identification of ions is based on the changes in ion mobility described by parameter *α*, defined by the following key formula [32]:

(1) $$K\left(E\right)=K_{0}\left(1+\alpha \right)$$

where *K*(*E*) is the ion mobility coefficient in the changed electric field, *K*_0_ is the reduced ion mobility coefficient (cm^2^ V^−1^ s^−1^), and *α* is the parameter identifying the proper species in the analyzed sample.

The quality of the spectrometer, as estimated by resolving power, is defined by the following equation:

(2) $$RP=\frac{CoV}{FWHM}$$

where *CoV* represents the compensation voltage of the center of the observed peak, and *FWHM* is the peak width in volts at half height [31].

In our research we used high-purity (>99%) air (dried) and nitrogen obtained from the Messer Group. The high-purity test VOCs were obtained from Merck (Rahway, NJ, USA).

**Figure 3:**
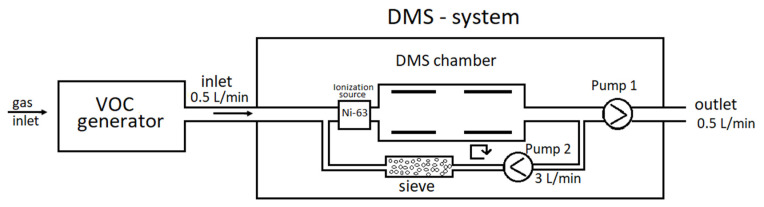
Diagram of the measurement system.

**Figure 4:**
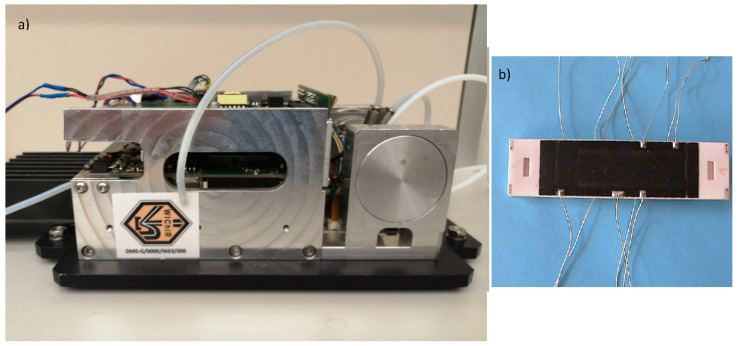
(**a**) DMS spectrometer; (**b**) ceramic DMS chamber.

DMS detectors were constructed at the Military Institute of Chemistry and Radiation (Warsaw, Poland) (Figure 3 and Figure 4).

First, 2 mL of liquid VOC samples were placed in glass vessels, which were placed in a stainless steel evaporator. The evaporator was thermally stabilized (range of stabilized temperatures: −10 °C to +90 °C). Next the VOCs’ vapors were diluted 1–100 times depending on the concentration needed. The concentrations of VOCs in the gas sample were controlled by a dual-FPD gas chromatograph type 8890 FPD (Agilent Technologies Inc., Santa Clara, CA, USA). The excess gas generated was directed to the exhaust hood while the DMS spectrometer collected the necessary amount of gas for analysis. Gaseous samples were taken from the cylinder and then diluted with nitrogen or purified air and fed to the DMS spectrometer; as before, excess gas was directed to the exhaust hood.

The purified air flew through the evaporator; its flow rate was regulated using the Bronkhorst mass controller (regulation range: 0–10 L/min). The employed flows were between 0.1 and 3.0 L/min. The second, purified air stream was connected to the evaporator’s exhaust. The second flow was regulated using the same mass controller. In this case, the flows were between 0.1 and 10 L/min. Taking advantage of two regulated streams (the first one through the evaporator, the second one acting as a diluter) and the VOC evaporator, which can be cooled, enabled us to work with a broad range of VOC concentrations. Specifically, the tested VOC concentrations were as low as ppb and as high as dozens of ppm.

Parameters of the control unit for the DMS spectrometer:

- High-speed, high-voltage (SV) generator frequency: 3 MHz;
- Selective voltage (peak-to-peak): 13–154 Td;
- Compensation voltage: −5.2 to 1.4 Td; 10 Hz;
- Maximum electric field intensity: 50 kV/cm;
- Length of DMS electrodes: 25 mm;
- Length of DMS chamber: 50 mm;
- Distance between DMS electrodes: 0.25 mm;
- Gas flow rate through detector: 3 L/min;
- Detector temperature: 45 °C;
- Ionization: Ni-63.

The aim of the following experiments was to test the detection capabilities of the developed DMS spectrometer, determining the detection limits for the most common VOCs and the possibility of identifying the detected substances.

For our experiments, we used our own DMS design. This is not a commercial spectrometer, but rather an instrument that is still under development and improvements at our institute. We intend to change the design to increase the spectrometer’s sensitivity and utility for the analysis of mixtures. The obtained results confirm the appropriate direction of this design.

## 3. Results and Discussion

A large group of chemical compounds are known as volatile organic compounds. Within this group, there are compounds with different chemical characteristics, including simple n-alkanes, highly toxic aromatic compounds such as benzene, toluene, and xylenes (BTX), as well as alcohols, esters, ketones, and chlorinated and fluorinated organic compounds. Due to their high volatility, these compounds can be analyzed with a DMS detector. Gas mixtures were prepared in special evaporation chamber, where the tested VOC vapors were mixed with a carrier gas. The data presented herein were collected over the course of several years of our research.

### 3.1. Detection and Identification of n-Alkanes

**Figure 5:**
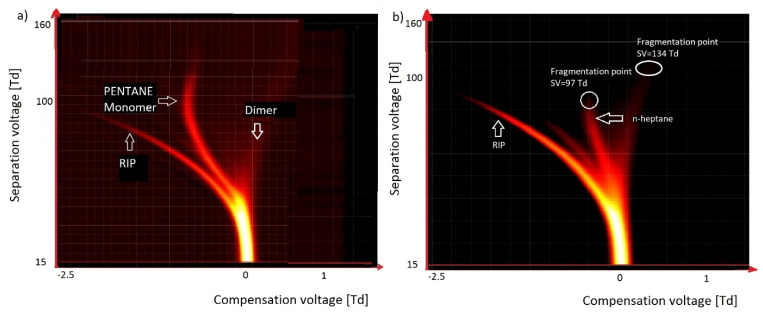
Dispersion plots for (**a**) pentane and (**b**) heptane.

The first group of compounds listed as VOCs are n-alkanes. Preliminary experiments with n-alkanes were carried out using clean and dried air (dried with molecular sieves; water level below 20 ppm) as a carrier gas. Figure 5 presents the spectra obtained for n-pentane C_5_H_12_ (concentration: ~250 ppm; Figure 5a) and n-heptane C_7_H_16_ (concentration: ~50 ppm; Figure 5b).

In the case of n-pentane, we observed a stable monomer ion with a high amplitude and a dimer. For n-heptane, we observed a monomer that was fragmented in a reduced electric field of 97 Td. For pentane, the fragmentation point was at a much higher value of 138 Td.

For the longer hydrocarbons, such as n-decane, the fragmentation point is very low, at 47 Td; thus, for n-decane, we could not see the fragmentation point—we could only observe fragmentation elements.

**Figure 6:**
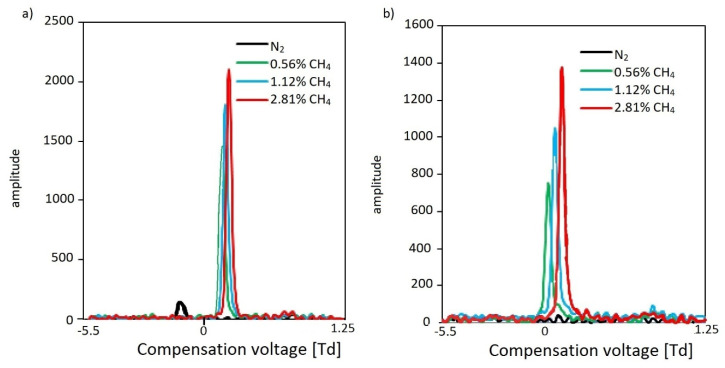
Differential mobility spectra for negative methane ions for SV voltage: (**a**) 115 Td; (**b**) 128 Td.

In the case of lower alkanes such as methane or ethane, analyte peaks were not observed when dried air was used as a carrier gas. This observation results from their having lower proton affinity (PA) values than that of water (691 kJ/mol), which for the simplest alkanes are as follows: methane, 534.5 kJ/mol; ethane, 593.3 kJ/mol; propane, 625.7 kJ/mol; pentane, 662 kJ/mol [33]; and decane, 691 kJ/mol [34]. The obtained results clearly indicate that the application of a carrier gas other than dried air is necessary; the replacement of dried air with nitrogen proved this point (proton affinity 494 kJ/mol), enabling the detection of methane at the level of 0.05% (*v*/*v*), observed for both positive and negative ions. We show the results for negative methane ions in Figure 6.

For negative ions for IMS, we did not observe RIN reactant ion negative; mostly O_2_^−^(H_2_O)_2–3_), but for DMS we may observe this peak for medium and low reduced electric fields (lower than 120 Td, corresponding to 109 Td for DMS). Such phenomena were not often described by the researchers, many of whom observed very low concentrations of oxygen species. As it is not supported by an experiment, this is only a hypothesis. Because it is not a typical RIN, we marked it as nitrogen (N_2_).

The presented results for n-alkanes from C1 to C10 indicate that they are detected and identified with a relatively high detection limit for methane from several thousand ppm, but for the next alkanes, the detection limit decreases proportionally to the number of carbon atoms in the n-alkanes—for hexane, the limit of detection reaches 30 ppm.

### 3.2. Detection and Identification of Aliphatic Alcohols and Acetate Esters

**Figure 7:**
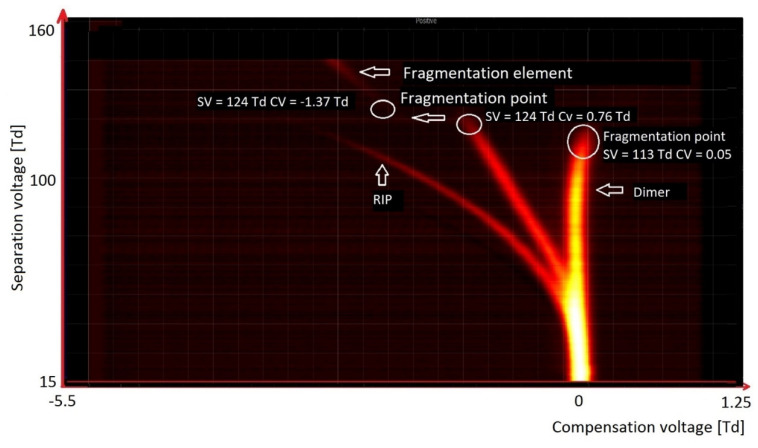
DMS spectrogram of ethyl acetate, with points indicating fragmentation.

**Figure 8:**
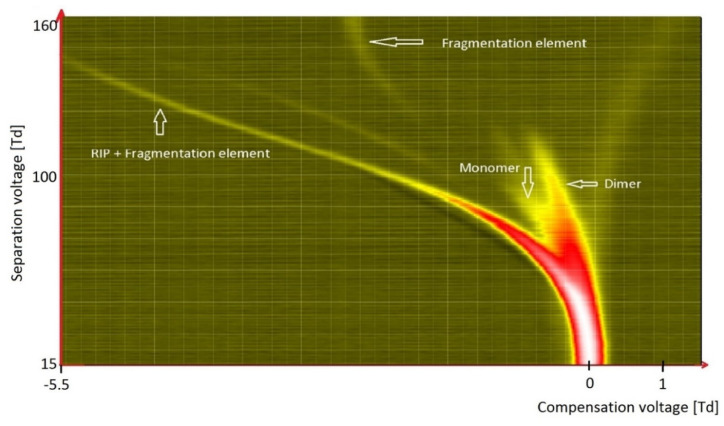
DMS spectrogram of ethanol, with identification of basic peaks.

Acetate esters and aliphatic alcohols belong to the group of compounds with a significant presence in the human environment. The DMS detector enables detection of these compounds with a very low detection limit (as low as several ppb). These compounds are connected, with a possible fragmentation even at very low ion energy and charge transfer [35]. Figure 7 presents the DMS spectrogram of ethyl acetate, while Figure 8 presents the DMS spectrogram of ethanol.

The identification of ethanol is more complicated compared to that of ethyl acetate, owing to the fragmentation of ethanol at lower voltage. One can correctly identify the kind of detected species only on the basis of primary peaks, before fragmentation. Fragmentation peaks only facilitate the identification of the analyzed compounds. Additionally, determination of the concentration is only possible based on the primary peaks, because only these peaks are concentration-dependent.

### 3.3. Detection and Identification of Ketones

The DMS detector enables the detection of ketones at the concentration level of a few ppb. Monomer and dimer peaks are formed for average concentrations, such as several dozen ppb. Very often, a dimer is subjected to fragmentation at a higher SV voltage. It is worth noting that ketones’ structure is similar to some extent to the structure of phosphoro-organic toxic compounds, which are also detected at a similar concentration level.

**Figure 9:**
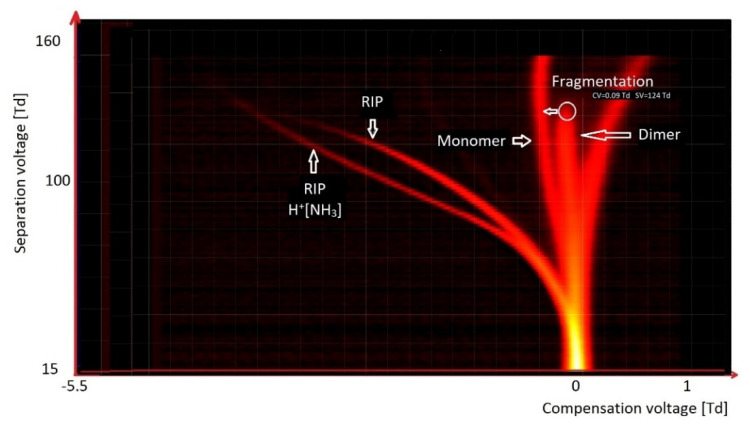
DMS spectrogram of octanone, with identification of basic peaks.

Figure 9 presents the spectrum of octanone. We observed two peaks of RIP (reactant ion positive; H^+^(H_2_O)_2–3_ and H^+^(NH_3_)). The other peaks originated from octanone. Over the entire voltage range SV, the monomer peak was stable. In the case of the dimer peak, it was stable up to 124 Td, and above this value it was fragmented.

**Figure 10:**
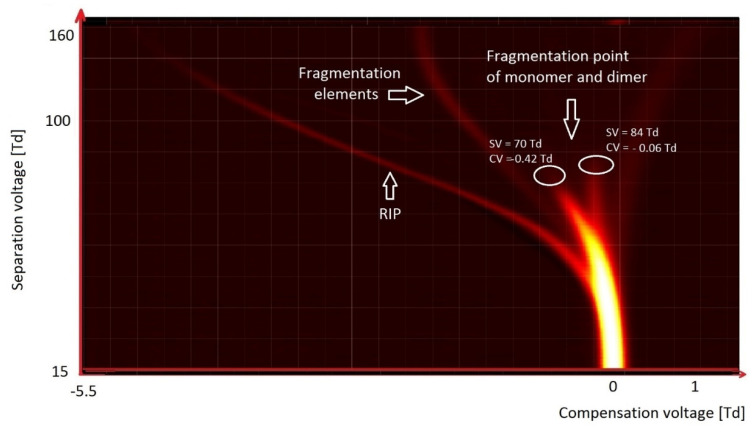
DMS spectrogram of acetone.

The simplest of ketones is acetone, presented in Figure 10. In the case of ketones, monomers and dimers are formed at low values of the reduced field strength. With an increase in the electric field strength—usually not higher than 200 Td—these ions disintegrate, and new ions are formed due to their fragmentation. Therefore, for acetone, ions decompose already at 70 Td, while for octanone they decompose at 124 Td. The reduced intensity of the electric field within which the decay occurs depends on the strength of the bonds in the molecule and, therefore, on its structure.

### 3.4. Detection and Identification of BTX Compounds

Benzene, toluene, and xylenes are VOCs, and are collectively referred to as BTX. Their analysis with a DMS detector was presented in the work of A. Szczurek et al. [36]; it was found that the detection limit for these compounds reaches as low as 0.5 ppm, but this is still too high a level, as the required detection level for these compounds is in the range of 0.01 ppm. This shows that even high PA values do not always guarantee detection at very low concentration limits. This is the case for BTX, where despite a high PA value (~750 kJ/mol), the compounds exhibit a lower detection limit than described for earlier ketones.

**Figure 11:**
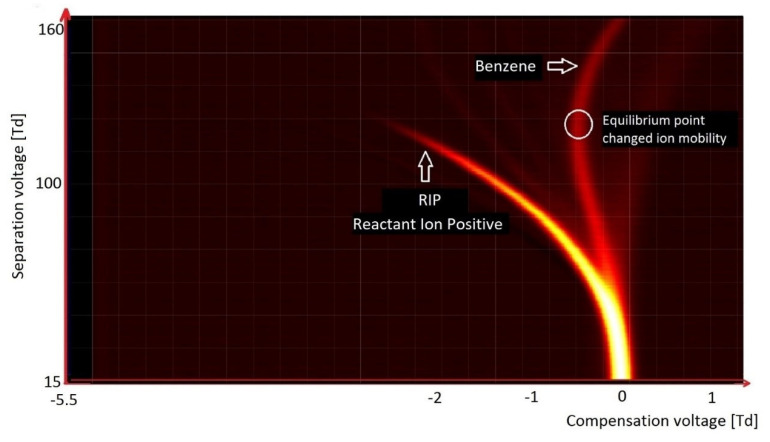
DMS spectrogram of benzene.

Only one monomer peak can be seen on the benzene spectrum (Figure 11). Monomeric ions move in an electric field in a very characteristic manner. Initially, the peak changes position in the direction of the lower CV values. After reaching an SV value of 115 Td, ions start to move towards the higher CV values. During our research, it was found that peaks from toluene and xylenes are located at practically the same position as those from benzene. The difference between the peak positions of benzene and the other BTX compounds is only to 0.22 Td. With the increase in the intensity of the electric field, the parameter *α* increases. This means that the ion mobility is increasing, and then after reaching an extremum position it starts to decrease. The extremum value for benzene is equal to 147 Td, for toluene it is 154 Td, and for xylenes it is 151 Td.

The observed change in the ion mobility for the BTX species may result from changes in the hydration of ion clusters, removal or addition of water molecules, or the increase in the effective ion temperature—that is, the sum of the thermodynamic temperature and some additional component resulting from the increase in the average energy of the ions under the influence of the electric field [37].

Characteristic of the spectrum of benzene is the presence of a deflection point (a shift of the peak in the opposite direction to the starting tendency). At the lower values of SV, the peak position shifts towards the lower values of CV; after reaching a certain value of SV, its further increase causes deflection towards the higher values of CV (Figure 11).

This deflection point, where these two processes are in equilibrium, is specific to a given compound, and can be used for its identification.

### 3.5. Detection and Identification of Chlorinated and Fluorinated Organic Compounds

**Figure 12:**
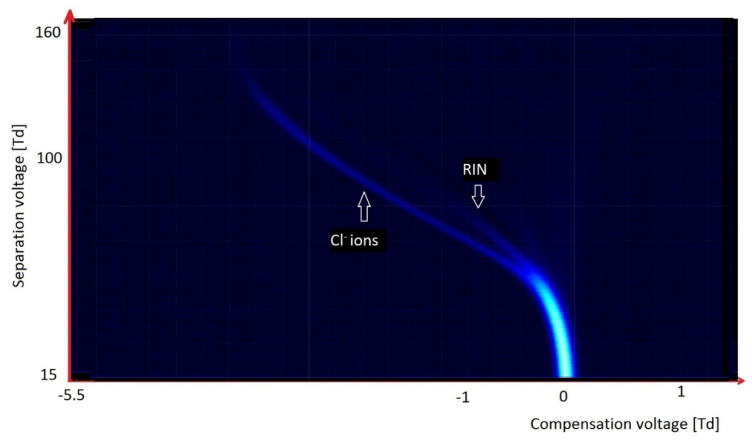
DMS spectrogram of tetrachloromethane.

Chlorinated and fluorinated organic compounds are readily ionized in a negative mode. With these compounds, detachment of chlorine and/or fluoride ions is easy. This often happens at the moment of ionization; it is visible on the spectrum obtained during the analysis of tetrachloromethane, CCl_4_ (Figure 12). Only two peaks are visible on the spectrum: from RIN, and the peak from Cl^−^ ions below RIN.

**Figure 13:**
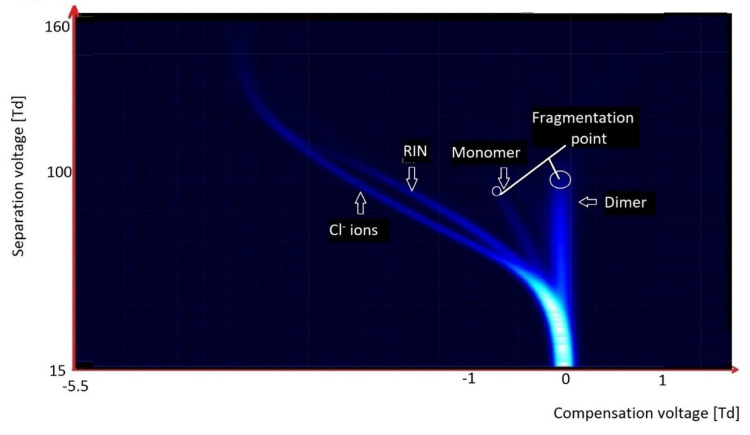
DMS spectrogram of hexachloroacetone.

In the case of hexachloroacetone (Figure 13), as was observed for CCl_4_, there were peaks originating from RIN and Cl^−^, but there were also monomer and dimer peaks from the fragmented compound.

In the case of halogen-containing compounds, we worked with the molecules exhibiting the lower electronegativity. Three kinds of ions can be formed: detachments of chlorine or fluorine (dissociative ionization); formation of particle ions; or formation of monomers and dimers (associative ionization).

On the spectrogram presented above, one can observe two kinds of ionization, i.e., separation of chlorine atoms, and formation of monomer and dimer species. This is a rather uncommon observation, because usually we can see only one kind of ionization [38].

In the case when a charge is well localized within the particle, peaks for monomer and dimer species are formed, and very high values of electric field—slightly above 100 Td (but below 150 Td)—are observed. This results in ion fragmentation, as can be seen on the spectrogram above.

When a charge within the molecule is not so precisely localized (benzene offers such an example), the shape of observed spectrogram is similar to the one representing BTX, and can be seen both for positive and negative ions.

### 3.6. Detection and Identification of Phenols

A characteristic feature of the chemical structure of phenols is the presence of the -OH group directly connected with the aromatic ring. This group activates the compound via electrophilic reactions. As a result of phenol ionization, the -O- group is formed, which due to its total negative charge donates more electrons than the simple -OH group. The hydrogen atom of the -OH group in phenols is cleaved more easily than in alcohols. Phenols are strong acids due to resonance stabilization of the anion.

Because of the presence of the hydroxyl group, phenols have a strictly localized charge. For compounds of this group, there is a strong detector response, already at the level of hundredths of a ppm.

**Figure 14:**
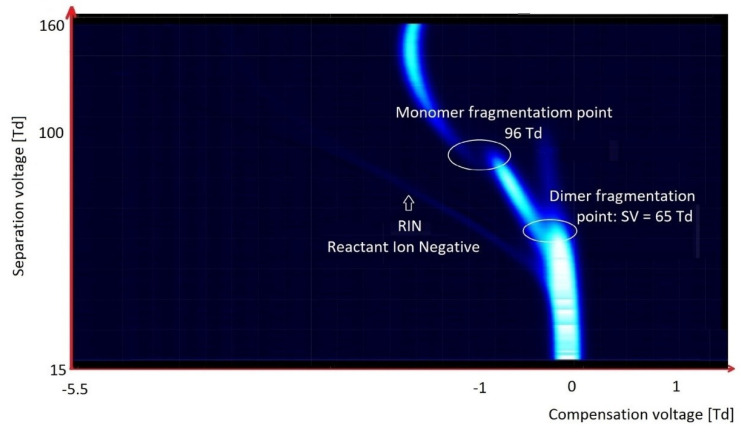
DMS spectrogram of phenol.

Figure 14 shows how DMS detects the phenol. In the spectrogram, we can see a few visible ranges of particle fragmentation; the first one is for the dimer, which fragmented to a monomer, while the next is for the monomer, which fragmented to an unknown ion. The second fragmentation suggests that these ions are benzene molecules, because they behave similarly to benzene molecules with an additional electron.

### 3.7. Detection and Identification of Psychoactive Species with the Differential Ion Mobility Detectors

**Figure 15:**
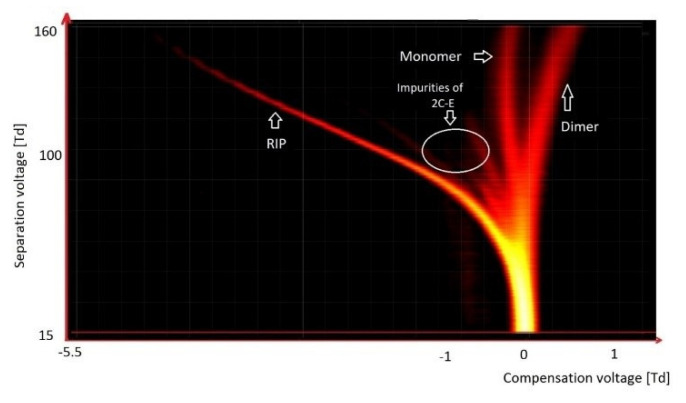
DMS spectrogram of 2C-E (phenylethylamine derivative).

**Figure 16:**
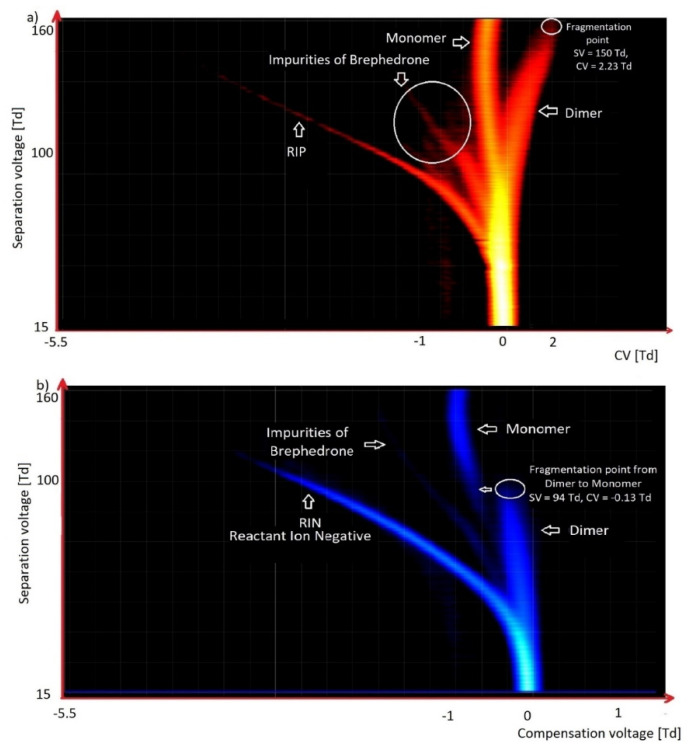
DMS spectrograms of brephedrone (1-(4-bromofenylo)-2-(metyloamino)-propan-1-on): (**a**) positive mode; (**b**) negative mode.

As outlined in the text above, multiple VOCs exhibit a relatively high vapor pressure. Consequently, significant amounts of these substances can be found in the gas phase, above the surface of the tested substance, and can therefore be detected using DMS. DMS detectors can also detect compounds such as psychoactive substances (drugs), exhibiting a much lower vapor pressure. Structures of these compounds are often very complex, which may contribute to their fragmentation in DMS; 2C-E (2,5-dimethoxy-4-ethylphenethylamine; a phenylethylamine derivative) is a typical example (Figure 15). Brephedrone is another characteristic substance for detection using DMS (Figure 16); this compound gives characteristic spectra in both positive and negative modes; the fragmentation proceeds in negative mode; however, since the concentration is very low, it is difficult to determine the spectrometer detection level.

**Figure 17:**
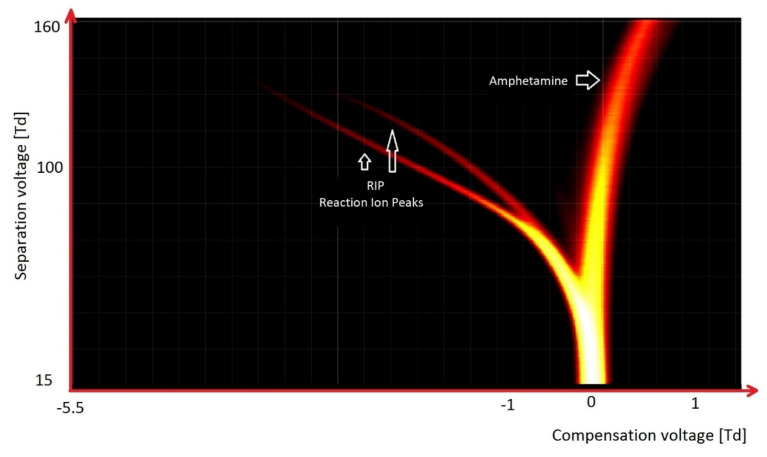
DMS spectrogram of amphetamine sulfate.

Among the most common psychoactive substances are amphetamine and its derivatives. The spectrum from DMS is an easy tool for identification and detection of these substances. Characteristics of this substance include the appearance of only one peak and the lack of ions in the negative mode (Figure 17).

It should be noted that when we used synthetic air as a carrier gas, we observe only RIP originating from H^+^(H_2_O)_2–3_ (Figure 10, Figure 11, Figure 15 and Figure 16a). However, when we used purified air (with molecular sieves and activated carbon), from the present traces of ammonia in the range of approximately 10 ppb we also observed an additional RIP peak H^+^(NH_3_) (Figure 9 and Figure 17). In the case of clean air, we observes only RIP and RIN peaks in the DMS spectra; when there were VOC contaminations, we observed additional peaks. The amplitude of these new peaks increases with the growth of VOCs’ concentrations, while the intensity of the RIP and RIN signals simultaneously decreases.

**Figure 18:**
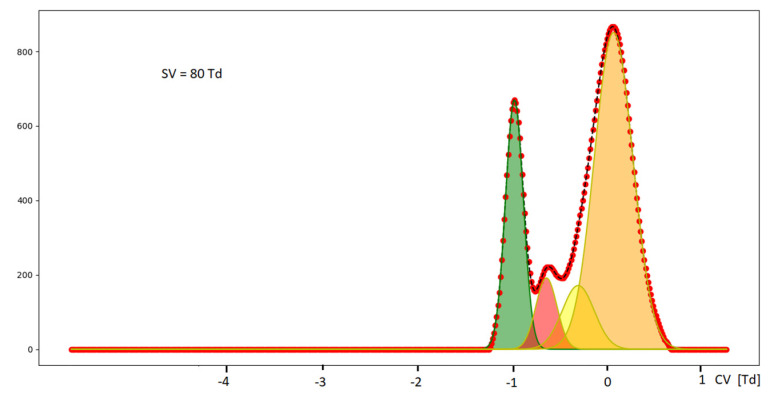
Results of the peak deconvolution program.

Changes in the spectrometer data analysis can be accomplished by deconvolution of peaks using algorithms that can be found, for example in the Python computer program [39]. Figure 18 presents results of this program, and a new peak can be detected.

In the case of the analysis of the fragmentation points, one can precisely find the analyzed compounds. Fragmentation points are determined based on defined CV and SV values.

Increasing resolution is possible via increasing the gap in the DMS chamber. For example, the increase in the gap from 0.25 to 0.5 mm, without changing the reduced electrical field intensity and gas flow, can double the resolution [31].

Increased sensitivity (lowering the detection limit) can be obtained via increasing the gap size while maintaining the same reduced field strength. When gap size increases from 0.25 mm to 0.5 mm, it is assumed that the sensitivity is doubled [40], which indicates that for the correct detection of BTX (at least two times below the NDS detection standard) the size of the DMS gap should exceed 0.6 mm. Additionally, it is recommended to detect BTX with high HSV values.

## 4. Ion Fragmentation: Effective Ion Temperature

Some of the chemical compounds during measurements with the DMS technique undergo fragmentation, similarly to the phenomena observed in mass spectrometry. In the case of mass spectrometry, the fragmentation of the molecules occurs during the ionization process; the substance is ionized with energies of 15–100 eV, which is a much higher level than the first ionization potential. Such high energy supplied to the molecules can cause each bond to break down and form fragment ions.

In the case of DMS, the voltage in the chamber increases, creating conditions for ion fragmentation. This is related to the ion effective temperature *T_eff_*, which is a measure of the energy of ions moving under the influence of the electric field. For the low values of electric field intensity, *T_eff_* is equal to the thermodynamic temperature *T*. When the energy of the electric field increases, the kinetic energy of ions gained during consecutive collisions with drift gas molecules has an impact on the *T_eff_* value. This phenomenon is described by the following formula [30]:

(3) $$\frac{3}{2}kT_{eff}=\frac{3}{2}kT+\frac{1}{2}M\nu_{d}^{2}$$

where *k* is the Boltzmann constant, *M* is the molecular mass of the drift gas, and *v_d_* is the velocity of the ion drift in the electric field.

Krylov [30] presented a formula that allows for the determination of the effective temperature on the basis of reduced mobility and the value of electric field intensity:

(4) $$T_{eff}\;\approx T+8.09\times 10^{-3}K_{0}^{2}{\left(\frac{E}{N}\right)}^{2}$$

where *K*_0_ is the reduced mobility in cm^2^ V^−1^ s^−1^, while *E*/*N* represents the reduced electric field in Td.

Based on Formula (4), the effective temperature for ethanol and ethyl acetate ions was calculated. It was assumed that *K*_0_ = 2.06, the fragmentation of the reduced electric field = 88 Td, the rectangular wave duty cycle = 30%, the drift gas was air, and the temperature = 45 °C. Based on these assumptions, the *T_eff_* fragmentation point for ethanol was ~290 °C and for ethyl acetate *T_eff_* was ~270 °C. During collisions with drift gas molecules, the ions only partly lose their energy, and the active energy field continues to effectively increase its energy. The energy increase depends on the strength of the electric field in the second power (4). However, with the increasing electric field energy, an increase in the number of inelastic collisions is observed. Introducing effect of the inelastic collisions requires the addition of a dimensionless coefficient *ζ*(*T*) to Formula (4) [41], which must be found experimentally. In the analysis presented here, this coefficient was skipped. If we assume that this coefficient is equal to 0.6, the effective temperature drops to 204 °C for ethanol and 192 °C for ethyl acetate.

It should be noted that the effective ion temperature is determined with limited accuracy.

The mobility of ions is described by the Mason–Schamp equation [33]:

(5) $$K=\frac{3q}{16N}\cdot {\left(\frac{2\pi }{\mu kT_{eff}}\right)}^{1/2}\frac{1+\alpha }{\Omega }$$

where *q* is the ion charge, *N* is the drift gas density number, *k* is Boltzmann’s constant; *T_eff_* is the effective ion temperature, *α* is the correction factor (less than 0.02), *µ* is the reduced ion mass, and *Ω* is the ion-neutral collision cross-section.

If we want to theoretically describe the curves of the spectrograms, we should know the effective temperature of the ions. However, because of the nonlinearity of *ζ*(*T*), it is not possible to obtain a formula showing changes in the peak position in relation to analyzed chemical species with an increasing reduced electric field.

## 5. Influence of Proton Affinity, Molecule Shape, and Charge Localization on the Detection Limit in Differential Ion Mobility Detectors

The ability of molecules to react to proton transfer is determined as proton affinity. The PA values for the discussed VOCs are displayed in Table 1 [33,34,42]. The data collected in Table 1 clearly indicate that the detection limit increases when PA values are high—equal to at least 700 kJ/mol. Detection of compounds with PA below 600 kJ/mol was impossible when the carrier gas is air. The detection of such compounds—including methane and ethane—was possible only with the use of nitrogen as the carrier gas. The detection limit for aromatic organic compounds decreases as much as 100-fold, compared to other compounds with similar PA values; this is related to the distribution of the charge within the molecule. In the case of aromatic compounds, the charge is delocalized in a cyclic manner, while in the case of compounds such as alkanes, for example, the charge is localized in one place.

In the case of attaching an electron to a molecule, we speak of electron affinity (EA). For EA, the same dependence occurs as for PA: a high EA value—as in the case of phenol 2.66 eV [43] or trichloromethane (chloroform) 2 eV [44]—and the location of the charge in the molecule allows for low detection limits.

## 6. Influence of the Electric Field on the Properties of a DMS Detector

Ions moving in the separation section oscillate between the electrode covers (Figure 1). The oscillation amplitude depends on the electric field strength, the interaction time for one phase of the voltage, and the mobility of the ions. If the ion path between the plates is negligibly small (as in the case of very high frequencies of the electric field), then the effect of reducing the peak amplitude by increasing the SV voltage is not observed.

When the ion paths during one phase of the SV voltage cycle exceed 20%, a further increase in the SV voltage will cause the amplitude of the detected peaks to decrease.

The second important parameter is the spectrometer resolution, i.e., the position of the peak in the spectrum relative to the half-width of the peak. For high SV values, peaks are better resolved; therefore, the SV value should be optimized to obtain the highest possible resolution with the lowest possible amplitude value.

Another aspect of the problem with very-high-frequency fields is ion fragmentation. In the case of DMS, ion fragmentation can cause a significant issue. As a result of fragmentation, an ion with very high mobility may be formed. In this case, the charge of the ion will be discharged by the control electrodes, or the charge will be transferred to other ions as a result of ion–molecular reactions.

For substances with very high fragmentation points (above 160 Td), the types of analyzed substances can be determined very precisely, because a large part of the VOCs are already fragmented, so the number of peaks for high SV voltages drops significantly; moreover, for high SVs, the resolution of the DMS spectrometer increases significantly. An increase in the reduced electric field from 70 Td to 150 Td constitutes a threefold increase in resolution.

One of the most important elements for a DMS spectrometer is the appropriate class of SV generator. For a gap of 0.25, the SV generator should operate at a frequency of 2 MHz and a maximum voltage of up to 1200 V, while for a gap of 0.6 (as recommended by the authors of this article), it should operate at a frequency of ~1 MHz, and the maximum voltage of the SV generator is up to 3 kV. The power consumed by such generators for the maximum voltage should not exceed 15 W. Maintaining such parameters for the DMS spectrometer enables its wide application for air monitoring.

## 7. Conclusions

Differential ion mobility spectrometry is a helpful analytical technique for easy, rapid (within seconds) detection and analysis of VOCs in the analyzed gas.

Thanks to DMS, people with dangerous materials such as explosives or drugs can be easily identified. However, the DMS technique suffers from poor analysis of mixtures. Higher resolution can be obtained by introducing changes in the construction of the DMS spectrometer, or in the data analysis methods. This can be achieved by increasing the spacing between electrodes up to the 1.5 mm range, and with the selection of an appropriate carrier gas flow rate. This allows for the reduction in the detection limits down to the single ppb levels. To ensure correct detection, the SV voltage should be maintained above 3 kV (100 Td) concurrently with a quasi-rectangular signal shape of ~30% duty cycle and a frequency of 1 MHz.

It can be concluded that the DMS has high development potential for low-level detection of VOCs, and can be used for permanent monitoring of air quality and detecting the presence of toxic substances.

## Figures and Tables

**Table 1:** PA values and DMS spectrometry detection limits for VOCs.

VOCs	Substance	Proton Affinity (kJ/mol)	Limit of Detection (ppm) (in Tested DMS Spectrometer)
n- alkanes	Methane	534.5; 523	Not detected in air
	Ethane	593.3; 561	Not detected in air
	n-Pentane	662	200
	Cyclohexane	686.9	30
	n-Hexane	678	15
	n-Decane	691	25
esters	Methyl acetate	821.6	0.01
	Ethyl acetate	835.7	0.01
	Propyl acetate	836.6	0.02
	Butyl acetate	~840	0.02
ketones	Cyclohexanone	841	0.01
	Acetone	812; 833	0.008
	Heptanone	845	-
alcohols	Methanol	754.3	RIP/RIN influence (10 ppm)
	Ethanol	776.4	0.015
	Isopropanol	786.5; 793	0.015
BTX	Benzene	754.4	0.5
	Toluene	784	0.8
	Xylene	796; 794.4; 812.1	0.6

## Data Availability

Not applicable.

## Sample Availability

Samples of the compounds are available from the authors.
